# The untargeted urine volatilome for biomedical applications: methodology and volatilome database

**DOI:** 10.1186/s12575-022-00184-w

**Published:** 2022-12-01

**Authors:** Maria Llambrich, Jesús Brezmes, Raquel Cumeras

**Affiliations:** 1 Department of Electrical Electronic Engineering and Automation, Universitat Rovira I Virgili, 43007 Tarragona, Spain; 2 Department of Nutrition and Metabolism, Metabolomics Interdisciplinary Group, Institut d’Investigació Sanitària Pere Virgili (IISPV), 43204, Reus, Spain; 3 Oncology Department, Institut d’Investigació Sanitària Pere Virgili (IISPV), 43204, Reus, Spain

**Keywords:** Urine, Volatilome, Metabolomics, Untargeted analysis, Biomedical, Methodology, Database

## Abstract

Chemically diverse in compounds, urine can give us an insight into metabolic breakdown products from foods, drinks, drugs, environmental contaminants, endogenous waste metabolites, and bacterial by-products. Hundreds of them are volatile compounds; however, their composition has never been provided in detail, nor has the methodology used for urine volatilome untargeted analysis. Here, we summarize key elements for the untargeted analysis of urine volatilome from a comprehensive compilation of literature, including the latest reports published. Current achievements and limitations on each process step are discussed and compared. 34 studies were found retrieving all information from the urine treatment to the final results obtained. In this report, we provide the first specific urine volatilome database, consisting of 841 compounds from 80 different chemical classes.

**Supplementary Information:**

The online version contains supplementary material available at 10.1186/s12575-022-00184-w.

## Introduction

Volatilomics is a branch of metabolomics that comprehensively analyses the volatile compounds released from biological samples. These compounds are products of metabolic processes in organisms, which are of great interest in clinical research [1, 2]. The volatilome constituents are endogenous when they are naturally produced by human metabolism or exogenous when they are produced by the interaction with an external exposure via inhalation, ingestion, or dermal absorption. A recent review has summarized the discriminant effect of the volatilome between different diseases and matrices [3]. Currently, up to 2,746 volatile compounds have been identified across 7 biofluids -breath, blood, faeces, milk, saliva, semen, skin, and urine- in healthy humans [4]. However, the accurate and complete composition of all volatiles that form the human volatilome is still unknown.

Urine is a complex matrix in terms of compounds as it contains metabolic breakdown products from foods, drinks, drugs, environmental contaminants, endogenous waste metabolites, and bacterial by-products. The benefits of using urine as a diagnostic biofluid are numerous, from the ease and non-invasive collection, easy storage and richness of compounds. Recently, over 400 volatiles have been identified in urine, belonging to more than 15 chemical classes, including hydrocarbons, carbonyl, carboxylic acids, and alcohols, among others [4]. Moreover, the urinary volatilome has been used to detect several pathologies and diseases, such as cancer [2], and tuberculosis [5], among others [1].

Measurement of the urinary volatilome usually requires a pre-concentration step to enhance sensitivity, traditionally based on the sample’s headspace (HS) fraction. Some of the techniques used are needle-trap [6], e-nose technologies [7], headspace sorptive extraction [8], thermal desorption sorbent tubes (TD) [9], and solid-phase microextraction methods (SPME) [10]. Microextraction techniques are the evolution of traditional liquid–liquid or solid–liquid extraction techniques, which require less sample volume and solvent [11]. The miniaturization of the pre-concentration techniques has opened new possibilities in medical diagnosis and improved the analytical limits. Among them, SPME has gained high popularity for its simplicity, sensitivity, and cost. Introduced by Pawliszyn et al. in the 90 s, SPME allows the concentration and extraction of sample compounds in a single solvent-free step [12]. Since then, SPME has become a popular method, and the methodology has been applied to multiple matrices with a wide range of purposes, from disease’s biomarkers identification to forensics studies [13–15]. Another emerging technique is the so-called needle-trap device (NTD), an evolution of the purge-and-trap method designed to detect trace organic compounds. In this case the sample is drawn inside the needle [16]. Despite the similar characteristics, NTDs devices have an exhaustive character, being able to deal with larger volumes. The initial NTDs were based in gas samples, although now it is used in a wide range of matrices like water, breath, or urine [14, 17]. Thermal desorption tubes are sampling methods based on diffusion; the thermal tubes sorbents have high hydrophobic properties reducing the interference of water—especially suitable for humid samples—but capturing a wide range of volatile compounds [18].

Microextraction techniques are based on two steps; first, the sorption of volatile headspace compounds to a stationary phase or sorbent; then, thermal desorption of the retained compounds. For its automatic and convenient sample introduction, microextraction methods achieve the highest potential when linked with gas chromatography-mass spectrometry (GC–MS) [19]. Although microextraction methods can also be linked to liquid chromatography-mass spectrometry, this is not easily accomplished in a single step [20]. Thus, high-throughput technologies, such as gas chromatography-mass spectrometry (GC–MS), have the advantages of robustness, high separation capability, sensitivity, and reproducibility [21]. Resolving power between peaks depends on gas chromatography equipment and methods, which can be improved using hyphenated techniques like comprehensive gas chromatography coupled to mass spectrometry (GCxGC-MS). The GCxGC-MS consists of two columns connected in a serial configuration where a modulator transfers the sample portions on the first column to the second one. All these advantages are highly desirable for the simultaneous detection and identification of compounds in complex matrices [22].

Analysis in volatilomics studies can follow targeted or untargeted strategies. Targeted analysis focuses on the extraction, determination, and quantification of specific volatiles of interest using a methodology optimized for that purpose. In contrast, untargeted strategies (fingerprinting) aim to identify the maximum number of volatiles in a sample. These untargeted strategies rely on the use of extraction and determination methods suitable for a broad range of volatility and polarity of volatiles. Then compounds are identified by matching each peak data with existing spectral libraries, followed by data analysis for biological relevance based on statistical methods [23]. Finally, driving the discovery of metabolomic patterns related to diseases.

**Fig. 1:**
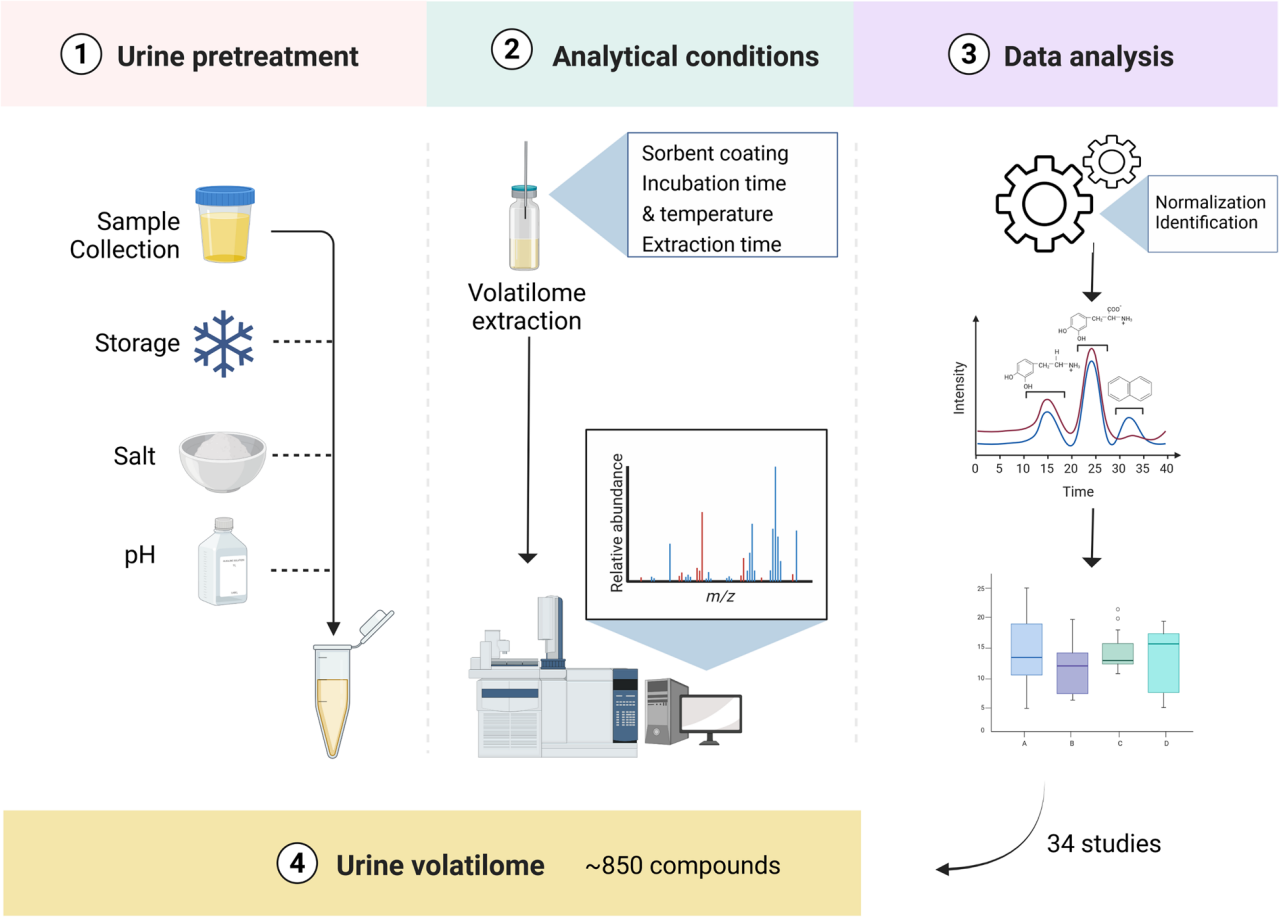
Schematic workflow for untargeted urinary volatilome methodology. The workflow steps include: 1) Urine pretreatment, including collection and storage conditions, and matrix modifications to enhance the extraction of the volatile compounds; 2) Analytical conditions, including the selection and tuning of several parameters from extraction parameters, sample incubation, and analytical instrumentation; 3) Data analysis including normalization and identification sources. 4) Urine volatilome obtained after combining results of the 34 studies analysed. Created with BioRender.com

This review focuses on untargeted volatilomics analysis of urine for biomarker discovery in diseases. The key elements of the untargeted workflow methodology are reviewed in detail (see Fig. 1), including: inherent urine matrix needs (collection, storage, and enhancement of compounds volatility); analytical procedures (extraction technique, optimal GC column); and data analysis (normalization, compound ID, etc.). Finally, the applications derived from the urinary volatilome are summarized, and a urinary volatilome database is provided (Table S1).

## Studies inclusion

The selection of the reported studies was done via PubMed, Web of Science, and Scopus search using the keywords “VOCS OR ‘volatile organic compound’”, “urine OR urinary”, and “human” and “GC–MS OR ‘proton transfer’”. Until May 2022 we gathered 230 studies (after duplicates exclusion). From those, after the title and the abstract screening, only the studies that conducted an untargeted analysis of human urine were considered. We discarded studies not involving urine (106 studies), no humans matrix (10 studies), targeted approach (41 studies), reviews or book chapters (24 studies), new materials (6 studies), or not disease related (9 studies). Therefore, a total of 34 articles with biomedical applications and have been assessed and included in the biomedical urinary volatilome database (see Table S1). All the 34 studies are GC–MS based, even though we considered proton transfer reaction mass spectrometry, but the studies in this detector kind were not about biomedical application in urine volatiles.

## Urine pretreatment

Collection and storage of urine are of paramount importance to preserve the volatilome as urine composition varies between day times. There are many approaches to urine collection, being the most used one spot urine or timed (many times during 24 h or shorter periods). The use of 24-h urine has the lowest day-time variability, but it is inconvenient for the volunteers; in such cases, the variability can be minimized using shorter periods for timed or one-spot collection with pre-analytical normalization methods [24]. Morning urine collection prevents variation due to external factors like physical activity [25]. Liu et al. tested the differences between morning sampling times, being the second-morning urine preferred for biomarker studies as it contains lower levels of dietary metabolites than first-morning urine [26]. In that sense, for volatilome analysis with clinical applications, the best option for sampling is second-morning urine, which includes overnight fasting by-products, but the dietary compounds are minimized. In Table S2 are summarized the different conditions of urine pretreatment by the 34 studies analysed.

Once collected, sample storage should maintain the volatilome. Urine can contain cells or bacteria that break upon freezing, thus pretreatment steps are used to remove them by centrifugation, filtering, and/or protein precipitation [24]. But centrifugation at high speed can cause cell breakage, provoking alterations in its volatilome [25]. Hence, direct aliquoting of samples is the preferred option to preserve the volatilome integrity, as it minimizes sample manipulation and the loss of volatile compounds. For measurements on the same day of collection, urine should be stored at 4ºC. For longer storage times, urine must be freeze. Some studies found that storage of samples at -80ºC was the condition that best preserves the compounds compared to fresh samples as no statistically significant differences were found between both conditions [27]. Storage for long times at -20ºC causes a considerable reduction in the amount of volatiles compared to the fresh condition [24, 27]. Another concern for the volatilome is the freeze–thaw cycles, Semren et al. showed that more than two cycles of freeze–thaw in samples stored at -80ºC influence significally the number of compounds detected [27].

There is a limited quantity of compounds in the headspace of urine. Therefore, to improve its extraction, it is necessary to enhance their affinity for the gaseous phase over the liquid phase. The most used method to enhance compounds to headspace in urine is based on salt addition, which produces a variation in the equilibrium where neutral organic compounds move to the gas phase [28]. Studies that tested the salt addition found an increase in the number of compounds extracted in the headspace[27, 29–32]. Nevertheless, salt saturation can be contra-productive due to opposite effects: a decrease in the number of compounds [30, 33], and an increase in the degraded compounds [34]. There is homogeneity between the analysed studies in choosing the same salt, sodium chloride (NaCl), in the range of 0.1 mg to 0.6 g NaCl/mL. Another alternative to increase the volatility is pH modification. The average normal urine pH is around 6, and its acidification (pH around 2) will increase the number of extracted compounds, mainly acids and sulfurs [34, 35]. On the other hand, the basification of urine (pH around 12) will show a limited increase in compound extraction, favouring mainly alcohols and heterocyclic compounds [30, 36]. The processing of samples with pH modification should consider the possibility of new compound formation due to side reactions of acidic media at high temperatures [30]. Considering the number of identified compounds as the figure of merit, the best performance is obtained (> 227 compounds identified) when combining all strategies (salt addition, acidic and alkaline pH) [36]. Even though it will be necessary to analyse the sample twice, one for acidic and another for alkaline conditions (see Table 1). Nevertheless, to maximize the results of a single measurement the urine pretreatment should include salt addition and acidification, only if the user needs to favour acidic compounds.

**Table 1:** Summary of included studies

	Study design		Sampling			Analytical conditions				Post-analysis	
Ref	Application	Sample size/ study design	Storage temperature	Volume sample/ vial	Pretreatment	Sorbent coating Temperature/ Incubation time/ Extraction time	Desorption Temperature/ Time	Instrumentation / Column phase	Analysis software/ Identification (match factor)/ RI	Normalization	Metabolites ID (statistically significant)
Cancer											
[37]	leukemia, colorectal and lymphoma	33 cancer vs. 21 controls	-80ºC	4 mL/ 8 mL	Salt + Acid	CAR/PDMS 50ºC/-/60 min	250ºC/6 min	SPME-GC-qMS/ PG	﻿Agilent MS ChemStation/ NIST05 (> 0.8)/ Yes*		82 (6)
[31]	Breast cancer (BC)	30 BC patients vs. 41 controls	-80ºC	4 mL/ 8 mL	Salt + Acid + IS	CAR/PDMS 50ºC/-/75 min	250ºC/10 min	SPME-GC-qMS/ PG	﻿Agilent MS ChemStation/ NIST05 (> 0.8)/ No*		116 (10)
[38]	Breast invasive ductal carcinoma (IDC)	65 IDC vs. 70 controls	-80ºC	4 mL/ 8 mL	Salt + Acid	CAR/PDMS 50ºC/-/60 min	250ºC/6 min	SPME-GC-qMD/ PG	Agilent MS ChemStation/ NIST11 (> 0.80)/ Yes	Quantile	94 (14)
[6]	Breast cancer (BC) + Colorectal cancer CCRC)	30 BC + 30 CRC vs. 60 controls	-80ºC	4 mL/ 20 mL	Salt + Acid	DVB/CAR1000/CARX 50ºC/40 min/4 min	250ºC/ 0.5 min	NTD-GC-qMS / OG	Agilent MS ChemStation/ NIST05 (> 0.80)/ Yes	Median	130
[39]	Prostate cancer (Pca)	58 Pca vs. 60 controls	-80ºC	1 mL/ 10 mL	IS	DVB/CAR/PDMS 44ºC/11 min/30 min	250ºC/5 min	SPME-GC-qMS/ BDPDP	Mzmine 2 software/ NIST14 / Yes*	Total area	122 (31)
[32]	Prostate cancer (Pca)	20 Pca vs. 30 controls	-21ºC	3 mL/ 10 mL	Salt	PDMS 50ºC/20 min/20 min	250ºC/4 min	SPME-GC-qMS/ 5DPDP	R dtw package/ -/-		n.a. (15)
[40]	Prostate cancer (Pca)	59 Pca vs. 43 controls	-20ºC	0.75 mL/n.d	Base	CAR/PDMS 60ºC/30 min/20 min	220ºC/5 min	SPME-GC-qMS/ 6CPDP	AMDIS & R metab/ NIST02 (> 0.8)/ No		n.a. (4)
[41]	Clear cell renal cell carcinoma (ccRCC)	75 ccRCC vs. 75 controls	-80ºC	2 mL/ 10 mL	Salt	DVB/CAR/PDMS 44ºC/11 min/30 min	250ºC/4 min	SPME-GC-qMS/ BDPDP	Mzmine 2.52/ NIST14/ Yes*	Total area	101 (11)
[42]	Renal cell carcinoma (RCC)	22 RCC vs. 25 controls	n.a	2 mL / 20 mL		CAR/PDMS 40ºC/-/40 min	200ºC/2 min	SPME-GC-qMS/ 5DPDP	Shimadzu GC–MS Post Run & XCMS/ NIST11/ No	Total area	n.a. (14)
[43]	Renal cell carcinoma (RCC)	30 RCC vs. 30 controls	-80ºC	2 mL/ 10 mL	Salt + Acid	DVB/PDMS 68ºC/9 min/24 min	n.d	SPME-GC-qMS/ 5DPDP	Saturn Workstation/ NIST14/Yes*	PQN + scaled to unit	181 (21)
[44]	Head and neck squamous cell carcinoma (HNSCC)	53 HNSCC vs. 82 controls	-20ºC	10 mL/ 20 mL	Salt + Acid	CAR/PDMS 50ºC/-/30 min	250ºC/6 min	SPME-GC-MSD/ 6CPDP	﻿Agilent MS ChemStation/ NIST08 (> 0.8)/ No	Mean peak area	81 (35)
[45]	Head and Neck cancer (HCN)	29 HNC vs. 31 controls	-80ºC	4 mL/ 8 mL	Salt + Acid	CAR/PDMS 50ºC/-/60 min	250ºC/6 min	SPME-GC-MSD/ PG	Agilent MS ChemStation/ NIST11 (> 0.75)/ No	Median & cube root	110 (28)
[36]	B-cell non-Hodgkin's lymphoma	101 lymphatic vs. 30 controls	n.a	10 mL/ 20 mL	Salt + Acid / Base	CAR/PDMS 40ºC/30 min/30 min	250ºC/n.d	SPME-GC-qMS/ 5DPDP	Shimadzu GC–MS Post Run/ NIST05/ No*		227 (3)
[46]	Non-Hodgkin lymphoma	18 Non-Hodgkin lymphoma vs. 40 controls	-80ºC	1.47 mL/ n.d	Salt	DVB/CAR/PDMS 90ºC/15 min/15 min	250ºC/5 min	SPME-GC-qMS/ 55DPDP	Mass Hunter Quantitative Analysis & MS-DIAL/ Fiehn8 & NIST14 (> 0.8)/ No	﻿LOWESS	n.a. (28)
[47]	Lung Cancer (LC)	20 LC vs. 20 controls	-80ºC	0.1 mL/ 2 mL		DVB/CAR/PDMS 45ºC/10 min/50 min	240ºC/10 min	SPME-GC-TOF/ 5DPDP	MassLynx & XCMS/ NIST08/ No*		19 (9)
[48]	Lung Cancer (LC)	17 LC vs. 30 controls	-80ºC	4 mL/ 20 mL	Salt + Acid	DVB/CAR1000/CARX 50ºC/40 min/4 min	250ºC/ 0.5 min	NTD-GC-qMS / OG	Agilent MS ChemStation/ NIST05 (> 0.80)/ Yes	Median	
[49]	Bladder Cancer (BdC)	9 BdC vs 7 controls	-80ºC	20 mL / n.d		DB-1 25ºC/15 min/-	n.d	NeedleEx®-GC-qMS/DMPS	Matlab/ NIST/ Yes		12 (5)
[50]	Bladder Cancer (UBC)	96 UBC vs. 209 controls	-20ºC	0.9 mL / 10 mL	Acid	DVB/CAR/PDMS 60ºC/30 min/20 min	n.d	SPME-GC-qMS/ 6CPDP	AMDIS + R Metab/ NIST11 (> 0.8)/ No	Median, log-transform and auto-scaled	n.a.(10)
[51]	Colorectal Cancer (CRC)	58 CRC vs 38 controls	-80ºC	5 mL / 20 mL		C2-AXXX-5149 40ºC/10 min/10 min	250ºC/10 min	TD-GC–MS/n.d	TOF-DS software/NIST 11 (n.d.)/No		23
[52]	Colorectal Cancer (CRC)	18 CRC vs. 31 polyps vs. 34 controls	-80ºC	4 mL		TD w/Tenax® + Sulficarb TA 60ºC/30 min/ 2 min	n.d	TD-GC–MS/ 6CPDP	XCMS/ NIST(> 0.8)/No	Square root	64 (31)
[53]	Cervical Cancer (CC)	12 CC + 5 CIN vs. 10 controls	-80ºC	2 mL / 20 ml		PDMS 60ºC/30 min/20 min	270ºC/ 5 min	SPME-GC-qMS/ 5DPDP	Agilent MS ChemStation/ NIST14	Autoscaling	220
Exposure											
[54]	Presence of exogenous factors (tobacco and air pollutants)	47 smokers vs. 68 non-smokers	-80ºC	3 mL/ 20 mL	Acid / Base	CAR/PDMS 37ºC/-/45 min	290ºC/1 min	SPME-GC-MSD/ PP	﻿Agilent MS ChemStation / NIST08/ No*	Creatinine	108 (27)
[55]	Health impact of environmental pollutants in high contaminated areas	36 Land of Fires vs. 14 Valley of Sacco River	-80ºC	1 mL/ 5 mL	IS	CAR/PDMS 60ºC/-/15 min	250ºC/n.d	SPME-GC-qMS/ 6CPDP	Enhanced Data Analysis/ NIST14 (> 0.6)/ No*		164 (4)
[56]	Human Exposures to Pyrethroids using an Exposure Reconstruction Approach	28 exposed to Pyrethroids	-80ºC	1.5 mL / 75 mL		TD w/Tenax® TA 25ºC/-/24 h	260ºC/10 min	TD-GC–MS/ 5DPDP	n.d		28 (n.d.)
[57]	Smoking	4 smokers vs. 3 non-smokers	-80ºC	0.5 mL/ 5 mL	Salt + Acid	DVB/CAR/PDMS 40ºC/-/30 min	250ºC/n.d	SPME-GCxGC-TOF / 5DPDP	ChromaTOF LECO/ Wiley 275 (> 0.9)/Yes*		294
Nephrotic diseases											
[58]	Mesangial Proliferative Glomerulonephritis (MsPGN) and IgA Nephropathy	A) 15 MsPGN + 21 IgAN vs. 15 controls B) 15 MsPGN vs. 21 IgAN	n.a	n.d		CAR/PDMS 40ºC/-/30 min	200ºC/2 min	SPME-GC-qMS/ 5DPDP	XCMS toolbox / NIST11 (> 0.72)/ No	Total area	15 (14)
[59]	Minimal Change type Nephrotic Syndrome (MCNS)	38 MCNS vs. 15 controls	n.a	n.d		CAR/PDMS 40ºC/-/20 min	200ºC/2 min	SPME-GC-qMS/ 5DPDP	XCMS toolbox/ NIST11 (> 0.75)/ No		n.a. (6)
[60]	Chronic kidney disease (CDK)	27 CDK vs. 20 controls	-20ºC	10 mL/ 20 mL	Salt	DVB/PDMS 40ºC/30 min/45 min	230ºC/1 min	SPME-GCxGC-TOF/ PG	ChromaTOF LECO/ NIST11 (> 0.9)/No	Specific gravity	282 (15)
[61]	Idiophatic membranous nephropathy (iMN)	63 iMN vs. 15 controls	-80ºC	5 mL/ 10 mL		PDMS/ 60ºC/5 min/10 min	250ºC/n.d	SPME-GC-qMS/ 6CPDP	Shimadzu GCMS + XCMS/ NIST11/No		n.a.(6)
Other diseases											
[35]	Autism spectrum disorders (ASDs)	24 ASD children vs 21 controls	-80ºC	4 mL/ 20 mL	Salt + Acid / Base + IS + 1 mL H20	DVB/CAR/PDMS 40ºC/-/30 min	230ºC/25 min	SPME-GC-MSD/ PG	n.d./ NIST05 and Wiley07/ No*		75 (9)
[62]	Overweight in children (OW/OB)	21 OW/OB vs. 28 normal weight	-80ºC	4 mL/ 20 mL	Salt + Acid / Base + IS + 1 mL H20	DVB/CAR/PDMS 40ºC/30 min/10 min	240ºC/25 min	SPME-GC-MSD/ PG	n.d./ NIST05 and in-house library/ Yes		110 acid / 83 alkaline (14)
[63]	Psychological disorders	30 stress patients vs. 30 controls	-80ºC	5 mL/ 10 mL		DVB/CAR/PDMS 50ºC/5 min/45 min	250ºC/n.d	SPME- GCxGC-TOF/ 6CPDP	ChromaTOF LECO/ NIST11 (> 0.6)/Yes	PQN	512(4)
[5]	Tuberculosis	117 tuberculosis + 56 non-TB controls	-80ºC	2 mL/ 20 mL	Acid	HS 100ºC/50 min/1 min	105ºC/1 min	HS-GC-MSD/ Propietary	AMDIS/ NIST /No	Total area	n.d. (5)
[64]	Coeliac Disease (CD) / Irritable Bowel Syndrome (IBD)	27 CD vs. 20 IBD	-80ºC	5 mL/ 10 mL		PDMS 60ºC/5 min/10 min	250ºC/n.d	SPME-GC-qMS/ 6CPDP	n.d./NIST/ No		70

^*^Confirmation by standards when available

n.a.: not applicable; *IS* Internal standard, *GC-MSD* Gas chromatography-Mass selective detector, *GCxGC-TOF* Comprehensive gas chromatography – time of flight detector, *SPME* Solid phase micro-extraction, *NTD* Needle trap device, *LOWESS* Locally weighted scatterplot smoothing, *MSTUS* MS total useful signal, *PQN* Probabilistic quotient normalization, *PG* Polyethylene glycol, *BDPDP* 1,4-bis(dimethylsiloxy)phenylene dimethyl polysiloxane, *5DPDP* 5% Diphenyl—95% Dimethylpolysiloxane, *6CPDP* 6% Cyanopropylphenyl – 94% Dimethylpolysiloxane, *PP* Porus polymer, *35PMP* (35%-phenyl)-methylpolysiloxane

## Analytical conditions

Needle-based microextraction is based on the time of contact and the affinity between the sample and the extraction phase of the device. The selection of the extraction mode depends on the sample matrix, analyte volatility, and the affinity of the analyte to the coating [19]. For the measurement of the untargeted urine volatilome, headspace SPME extraction is the preferred mode based on the studies found. The urine HS-SPME extraction opens the possibility to obtain cleaner extracts, higher selectivity, and guarantee longer fibre coating life-time. Nevertheless, Needle-trap techniques or Thermal desorption are also selected to have a broad picture of the urinary volatilome. Table 1 summarizes other parameters involved in the extraction, such as partitioning, sorbent coating, extraction time and temperature, analytes transfer, and instrumental conditions. In Table S3 are summarized other relevant extraction parameters, like coating type, the volume of urine used, incubation time and temperature, extraction time and temperature, and if stirring was used.

The SPME fibre sorbent coating materials will determine the affinity of the volatile compounds [12]. Nowadays, manufacturers offer a broad range of coating chemistries with different selectivity (affinity to different compounds), being the most used for volatilome applications polydimethylsiloxane (PDMS), Carboxen/PDMS (CAR/PDMS), Divinylbenzene/CAR/PDMS (DVB/CAR/PDMS), and polyacrylate (PA). We compared the outcome from each clinical study, despite the differences between urine pretreatment conditions (like volume, salt, or pH), where the highest number of compounds extracted—227- were obtained with CAR/PDMS, followed by DVB/CAR/PDMS with 176 compounds. Other coatings, like PDMS or PA, extracted less than 60 compounds. Five of the studies included in this review have compared the performance of different fibres for urinary volatiles determination, being the CAR/PDMS fibre selected when samples were under acidic conditions [27, 29, 37]. In contrast, under acidic/alkaline conditions or with non-pH modifications, the choice is a DVB/CAR/PDMS fiber [30, 35]. When used a GCxGC-MS, the selected coating is also DVB/CAR/PDMS despite whether urine has acidification or not. Thus, combination of matrix modification – salt and pH – with CAR/PDMS coating disclose the best results for urinary volatilome determination.

The time and the temperature the fibre spends exposed to the sample headspace (the so-called “extraction time”) also affect the efficiency of SPMEs. In the studies, the extraction time ranges from 15 to 90 min, and the temperature ranges from 37ºC to 90ºC. The best conditions for urinary volatilome are obtained with an extraction of 30–45 min at 40-60ºC (see Table 1). Cozolino et al. reported the smallest temperature after their optimization method, detecting 75 analytes with an extraction condition of 30 min at 40ºC [35]. Nevertheless, when the same extraction parameters are analysed with GCxGC-MS, the number of identified compounds increases to 294. Silva et al. 2011, and Drabinska et al. obtained their best performance at 60ºC with 60 min and 45 min extraction times, respectively [30, 37]. Nonetheless, Silva et al. 2019 found an optimal temperature of 70ºC [31], but this high temperature caused fibre damage and sample degradation, in accordance with previous studies [33, 37].

Thermal desorption tube coatings have different affinities for the volatiles based on the coating’s combination, the most famous is the Tenax TA coating – porous polymer –. Tenax was used in all studied evaluated for TD, except for one study where Tenax TA coating was combined with another sorbent, Sulficarb TA – carbonised molecular sieve – [52]. Despite the differences in pretreatment conditions, the best conditions for TD tubes was when Tenax was combined with Sulficarb with 30 min extraction at 60ºC identifying 64 compounds [52]. Comparable results are observed using a 24 h incubation at 25ºC and 20 min incubation at 40ºC with 28 and 23 compounds, respectively [51, 56]. Another type of extraction is needle trap microextraction, two different coatings are evaluated. Divinylbenzene/Carboxen 1000/ Carbopack X(DVB/CAR1000/CARX) extracted 130 and 98 compounds. However, when Dimethylpolysiloxane (DB-1) was used only 12 compounds are identified [49]. Table S4 summarizes the relevant GC and MS conditions of the analysed studies.

In the final step, the analytes are transferred from the devices to the GC, known as the desorption phase. The device is placed in the GC injector at a high temperature for the complete transfer of analytes by desorption [65]. Studies about best desorption parameters range from 1 to 25 min and 200ºC to 290ºC. Only Song et al. compared different desorption times for SPME and found after optimization that the optimal desorption time was 5 min [29]. Similar ranges are used in all devices except for direct headspace, where the temperature was 105ºC and 1 min for desorption.

## Data analysis

The basis of all untargeted analysis is to identify as many compounds in a sample as possible to obtain a profile, in this case, the volatilome. For that, the strategy followed is based on the use of software, commercial or open-source, which includes all the analysis workflow from raw data to the list of identified compounds suitable for statistical analysis. More than half of the studies used commercial software for data processing, whereas the others used free software or open-source solutions, such as MS-DIAL [66]. The pre-processing pipeline includes peak detection, noise removal, deconvolution, alignment, and compound identification. Deconvolution separates overlapped peak signals, and then they are identified based on their spectra and elution time [67]. One strength of GC is the electron impact ionization (EI) source, which is generally used at 70 eV, considered a hard ionization since it completely breaks the compounds and produces reliable and reproducible patterns of their fragments. Thus, the independence of the patterns to the instrument allows an accurate peak identification by matching with open or commercial spectral libraries [68], especially if the Retention Index is also included.

Compound identification is a complex process, as with current tools is not possible to identify all the compounds detected in a sample. The reason lies in the identification process which is based on libraries or databases that are not yet completed because not all known metabolites can be purchased or even synthesized [69]. Moreover, not all libraries include RI, key information that allows a more secure and specific way to ensure proper identification. Here starts the user interaction. Once the peak table is obtained, the user has to select the minimum similarity factor between experimental and library spectra (usually cosine similarity) for the identification query, with a minimum acceptable value between 0.6 to 0.8 (see Table 1), based on different instrument and library conditions. The highest level of identification, metabolite standard level 1 (MS1), is achieved using two independent and orthogonal datasets, which is usually a confirmation using reference standard compounds [70], nominal mass spectra and retention index. But on a routinely untargeted analysis, the number of putative compounds identified is in the range of hundreds, and the confirmation by reference standard compounds of all of them becomes tedious and expensive (if not right away impossible). Nevertheless, in GC–MS the retention index (RI) achieves a MS1 level. RI is an orthogonal confirmation based on retention time (RT), a measure independent of experimental conditions and unique for each compound [71]. To obtain RI values, a set of compounds (aliphatic alkanes or FAMEs) are used as indicators [69]. Despite the increase in confidence that offers this measure, only 7 authors used the retention index (see Table 1). The applicability of RI in volatilome analysis has some limitations, as RI library values depend on the column polarity selected, and some of the volatile-specific columns used (e.g. ZB-624) have non-specific RI libraries. Moreover, in available commercial libraries, like NIST, only 11% of the compounds include the RI [72], which makes identification a limiting step.

The GC–MS profiles obtained after the data processing are tables containing the relative abundance of each peak detected: intensity, area, or both; its RI or RT and their identification. Comparative analysis uses this information to evaluate the differences between group samples and find compounds of interest [73]. Although this approach is used widely in metabolomics, it has considerable unwanted experimental and biological variation [74]. Experimental variation due to human error and instrument bias is corrected with the use of internal standards (IS). IS are compounds added in constant amounts to all samples, usually deuterated forms like 1,4-Dichlorobenzene-d4; or not biological compounds, like 4-Fluorobenzaldehyde [39, 55]. Notwithstanding their usefulness, only 25% of the studies reported the use of IS. Moreover, the biological variation in urine concentration is high, as it is a biofluid that is not homeostatically regulated, which can mask the variations due to internal factors. Compounds concentration in urine will depend on the hydration status of the individual. Thus, normalization becomes a fundamental step in metabolomics that is poorly implemented. However, only in 40% of the studies analysed it was somehow accounted for (see Table 1). Among the strategies used for normalization, there are several approaches like normalization by total area, creatinine, quantile, and median. The strategy most used is to normalize by total area: briefly, it is the division of the area of each peak by the sum of all the peaks’ areas. However, this strategy can mask variations due to differences in peak number as new peaks in samples are diffused across all the samples [73]. Similarly, in creatinine normalization, the peaks are normalized by the creatinine concentration of the sample. Creatinine concentration has been widely used in clinical applications as a urine normalization method. In the Human Metabolome Data Base (HMDB) [75], the urine compounds concentrations are reported normalized to the creatinine concentration. However, recent reports have proven that other factors such as diet, exercise, or gender, influence the excretion of creatinine [72]. Similarly, MS total useful signal (MSTUS) has been proposed as a normalization method, where the signal is divided by the sum of features common in all samples [76]. Quantile normalization refers to an intensity-dependent scaling factor and transformation of peaks [74]. Finally, median normalization is the division of the profiles by the median of all study profiles [73]. Other more statistically-oriented normalization methods exist [77], like the locally weighted scatterplot smoothing (LOWESS) algorithm (based on a local regression) or the probabilistic quotient normalization (PQN). Mack et al. compared 5 normalization methods in urine volatilome (creatinine, osmolarity, urine volume, MSTUS and PQN) [78]. All methods showed comparable results, but none of them could deal with problems in renal function. Urinary volatilomics for clinical research is still emerging in part because there is not a generally agreed standard normalization method yet, so the researcher must choose a determined strategy. Nevertheless, normalization methods for urine are highly established for other purposes. It is the case of epidemiological studies where is used the normalization by specific gravity [79, 80]. Specific gravity is used as pre-processing normalization method where the samples are diluted to the same concentration. But, there is only study using specific gravity applied as a sample selecting parameter [60].

## Applications

Urine GC–MS untargeted analysis has been applied to several clinical topics: cancer, harmful chemicals, and nephrotic diseases, among others (see Table 1 and Table S5). Cancer is the most studied disease as twenty-one studies evaluated biomarker discovery for a range of cancer types such as prostate, breast, or renal. Another topic of relevance is the study of harmful chemicals in humans, where four studies evaluated the effects of polluted environments and tobacco. By the direct relationship with the urinary system, nephrotic diseases are an interesting topic, where the urinary volatilome is used to improve the diagnosis of some nephropathies. Finally, diseases not included in the previous groups are classified as other diseases, including autism, overweight children, psychological disorders, tuberculosis and coeliac disease.

**Fig. 2:**
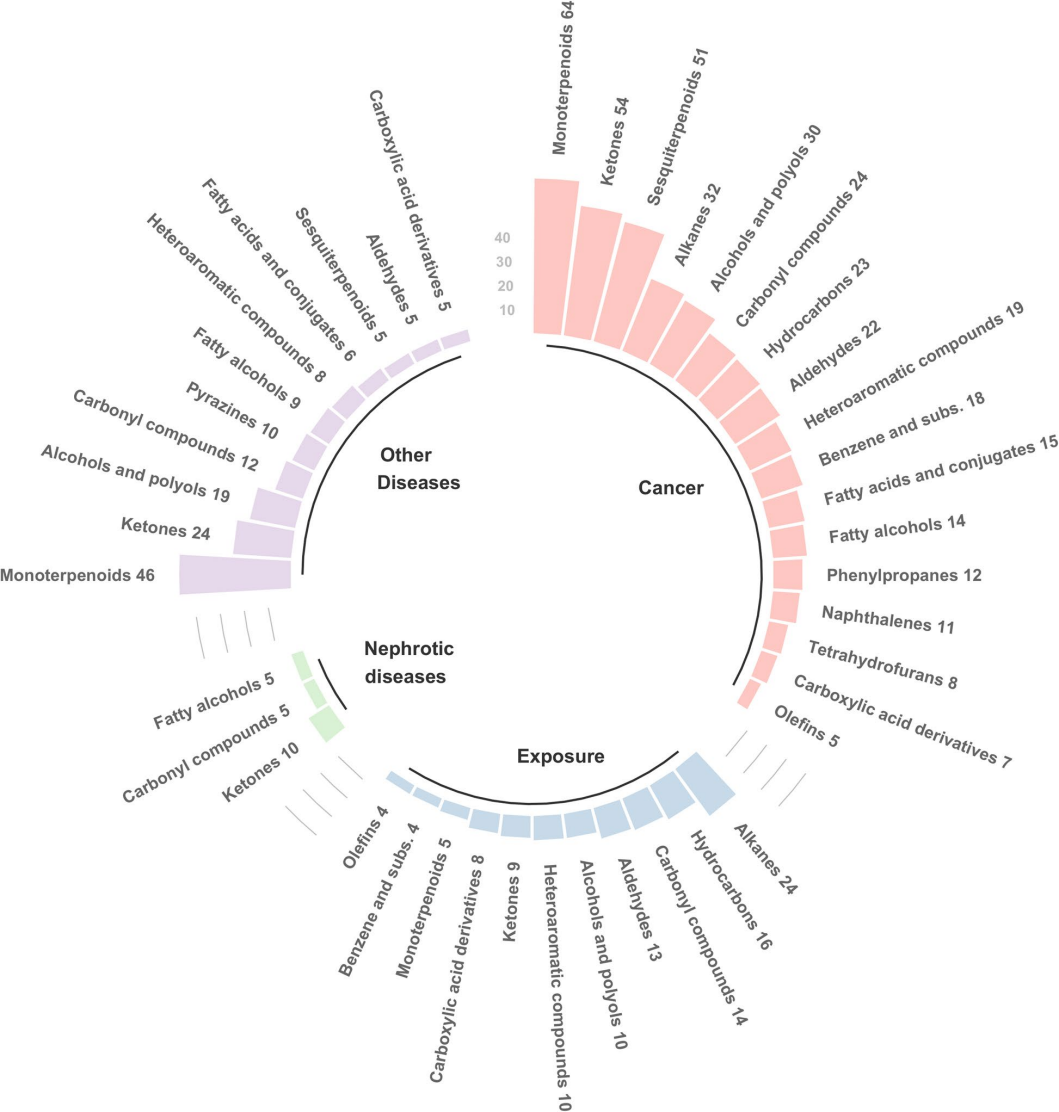
Circle bar plot for the chemical classes identified in human urine volatilome in biomedical conditions classified by application group, for the studies reviewed with a compound list disclosed. Studies included are divided into 4 groups by the application. The number of compounds within each class corresponds to the number of unique species identified for that chemical subclass

The biomedical untargeted urinary volatilome database includes the compounds identified in studies of biomedical applications using the urine volatilome; in total, we retrieved the information from 34 studies. One study on the cancer group was not included in the database creation, as they did not provide the compounds identification [32]. We retrieved and harmonised all detected compounds for each of the 33 included studies using the same InChIKey identifier [81]. The included studies reported 841 different volatiles (Table S1), of which 2-pentanone, and 4-heptanone were found in at least half of the studies (Table 2). From the urinary volatilome list, only 267 compounds were retrieved in two or more studies. The smallest compound detected is acetonitrile (C2H3N), whereas the biggest is Allyl octadecyl oxalate (C23H42O4). Given the complexity of comparing specific compounds per group, we performed the comparison by the chemical classes found within each group, which were retrieved with the ClassyFire tool [81]. The number of chemical classes found spans from 63 in cancer to 23 in nephrotic diseases. The chemical classes more present in all groups are organooxygen compounds, organic disulfides, and phenols. But with different abundance across groups. Focusing on subclasses, ketones are highly represented in all groups, being the more abundant in cancer. In contrast, the exposure groups have a higher abundance of alkanes, whereas the other diseases group is characterized by monoterpenoids (see Fig. 2). For each group, we performed an enrichment analysis. However, only for the cancer group, the enrichment analysis returned significant pathways (with *p*-values < 0.05 and false discovery rates FDR < 0.05).

**Table 2:** Compounds of the biomedical untargeted urinary volatilome database found at least ten times in the included studies (*n* = 33)

Nº studies	Compound	Chemical Subclass	HMDB ID
21	2-Pentanone	Ketones	HMDB0034235
18	4-Heptanone	Ketones	HMDB0004814
15	Hexanal	Aldehydes	HMDB0005994
14	Dimethyl disulfide	Dialkyldisulfides	HMDB0005879
13	2-Butanone	Ketones	HMDB0000474
13	2-Propanone	Ketones	HMDB0001659
12	2-Heptanone	Ketones	HMDB0003671
12	Nonanal	Aldehydes	HMDB0059835
12	Phenol	Phenols	HMDB0000228
11	Acetic acid	Carboxylic acids	HMDB0000042
10	3-Hexanone	Ketones	HMDB0000753
10	Carvone	Monoterpenoids	HMDB0035824
10	Furan	Heteroaromatic compounds	HMDB0013785

### Cancer

The main application in clinical research is cancer, as several authors have proven the usefulness of volatilome analysis in urine samples for biomarker discovery in this disease. B-cell non-Hodgkin’s lymphoma disclosed the higher number of compounds identified among the cancer types tested (227 compounds, as seen in Table 1). Mesquita et al. also evaluate Non-Hodgkins lymphoma finding 28 volatiles statistically significant [46]. Prostate cancer is one of the most studied, as it is evaluated in three studies using different approaches of SPME conditions and GC column phases. Khalid et al. identified 197 compounds with CAR/PDMS at 60ºC, Lima et al. identified 122 compounds with DVB/CAR/PDMS at 44ºC, and Deev et al. used PDMS at 50ºC but none of them performed compound identifications [32, 39, 40]. Similarly, 3 authors evaluate renal cell carcinoma, Monteiro et al., Pinto et al., and Wang et al. found 21, 11 and 14 compounds statistically significant, respectively [41–43]. All used SPME extraction but with different conditions, being the combination of DVB/PDMS at 68ºC the one with more compounds identified [43]. Head and neck carcinoma was evaluated by two authors using the same SPME fibre coating and time; Taware et al. identified 110 compounds, whereas Opitz et al. found 81 compounds [44, 45]. Conditions for breast cancer only differ in the SPME exposure time used: Silva et al. 2019 applied 15 min longer extraction time with 116 compounds identified; and Taunk et al., identified 94 compounds but they get higher number of compounds statistically significant [31, 38]. Similar conditions were used to evaluate a set of samples from leukaemia, colorectal cancer, and lymphoma. In this case, 6 compounds allow to differentiate between cancer and healthy patients. Lung cancer is usually studied through breath, but in two studies it was also evaluated using the urine volatilomics profile. The authors selected different methodologies; Hanai et al. used SPME extraction identifying 19 compounds whereas Porto-Figueira et al. used NTD to identify 98 compounds [47, 48]. Bladder cancer has a direct relation with urine, Jobu et al. evaluate it by NeedleEx whereas Lett et al. used SPME [49, 50]. Comparison of techniques is not possible as results are not completely disclosed. Colorectal cancer was analysed by thermal desorption by two authors differing in time and temperature of extraction, studies showed 12 and 23 compounds identified [51, 52]. Díaz de León-Martinez et al. evaluated the urine volatilome for cervical cancer with SPME obtaining one of the highest number of compounds identified -220- using only 2 ml of urine [53]. Discrimination between cancer patients and controls is possible with the use of urine volatilomics [82]. Together, with the elevated number of studies, the cancer studies’ group shows the highest number of volatiles classes detected. The chemical classes found in higher proportion are terpenoids and carbonyl compounds (including ketones and aldehydes). There are 31 chemical classes unique for the cancer group, the most abundant being tetralins, cinnamaldehydes, and lactones. Three compounds are found in at least half of the cancer studies analysed (see Table S6): 2-pentanone, phenol, and dimethyl disulfide.

**Fig. 3:**
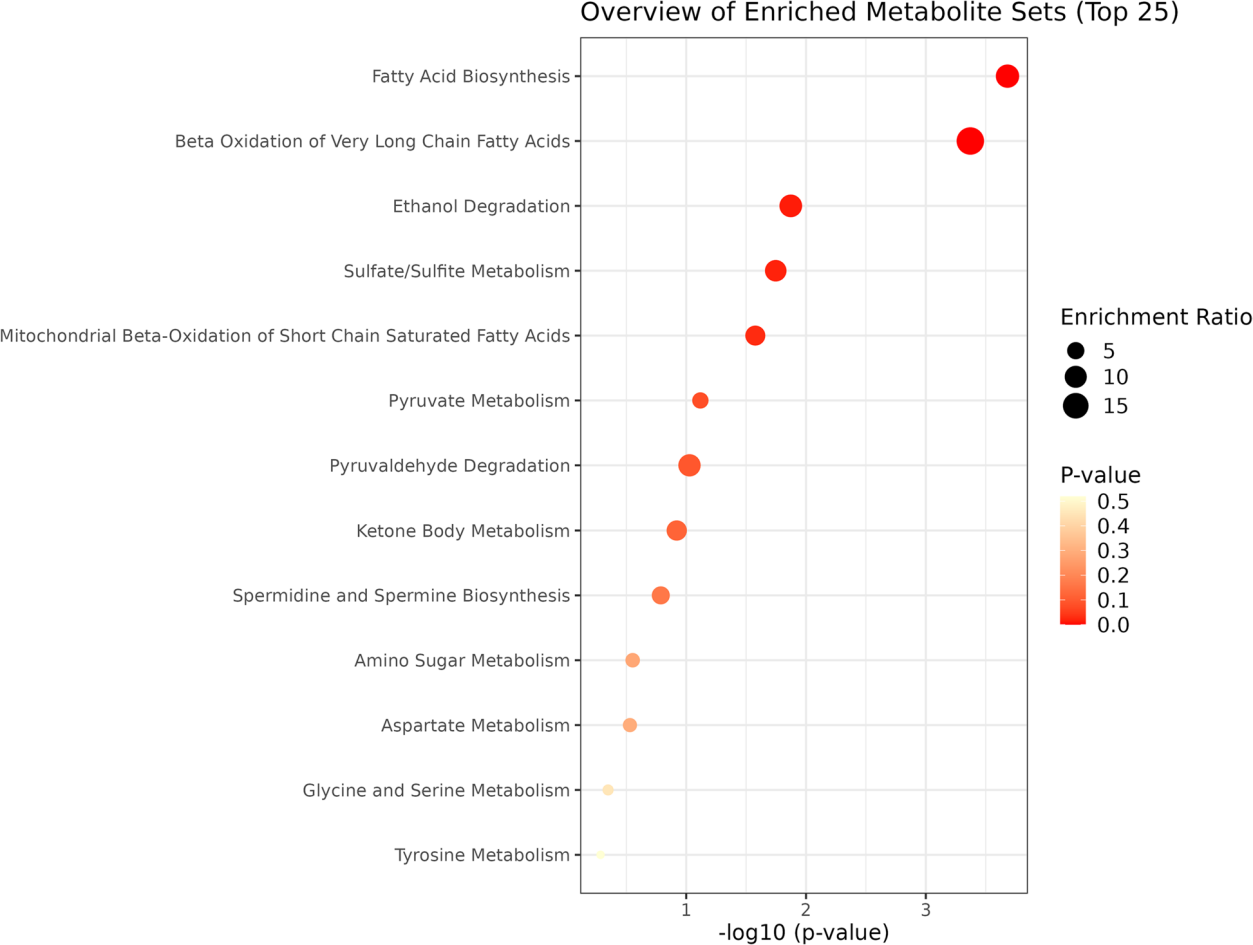
Dot plot of the enrichment analysis results for the cancer application. The size of the circles per metabolite set represents the Enrichment Ratio and the colour represents the p-value. Analysis performed with MetaboAnalyst Enrichment Analysis [83] module using identifiers from HMDB and KEGG

An enrichment metabolites analysis was performed to identify classes of metabolites that are over-represented in the large set of metabolites that conform the cancer group (641) and may have an association with cancer. Up to thirteen pathways are found to be over-represented (see Fig. 3), however, only the fatty acids biosynthesis and the beta oxidation of very long chain fatty acids are significant (*p*-value is 0.0002 and 0.0004, respectively and the FDR is 0.02 for both pathways).

### Chemical exposure

Urine is the biofluid most used to assess the exposure of some chemical compounds that may be harmful to humans, including third hand tobacco [84]. Longo et al. assess the fingerprint in urine for areas with high air pollution in Italy [55]. The comparison between two areas with different pollution identified 164 volatiles, where only 4 of them were statistically significant. Previously, Filipiak et al. assessed the potential of smoking and the environmental exposure in two biofluids, breath and urine, finding 108 volatiles in urine [54]. SPME conditions were different in both studies, but they used the same fibre coating (CAR/PDMS). When SPME is coupled to GCxGC-MS the number of compounds detected increase, as reported by Rocha et al. for smoking comparison with 294 compounds identified [57]. One author selected another extraction methods for exposure analysis, O’Lenick et al. studied the exposure to pyrethroids using thermal desorption with the identification of 28 compounds [56]. Exposure related compounds belong to 52 chemical classes. The chemical classes found in higher proportion are carbonyl compounds (ketones and aldehydes), alkanes and hydrocarbons, where 10 are unique for exposure applications, such as benzofurans, oxazinanes and azolines. One compound is found in all the chemical exposure studies (see Table S6): hexanal.

### Nephrotic disease

As part of the urinary system, some nephrotic diseases have been evaluated through the volatilome of urine. Biomarker discovery was successfully used in minimal change type nephrotic disease (MCNS), a disease with an invasive diagnosis that affects mostly children. Liu et al. identified 6 volatiles as possible biomarkers of MCNS [59]. In the same line, 5 volatiles were identified as possible biomarkers for mesangial proliferative glomerulonephritis [58]. Diseases may lead to chronic kidney disease (CDK), which was investigated by Ligor et al. using GCxGC-MS. The CDK patients showed a panel of 4 volatiles upregulated (methyl hexadecanoate, 9-hexadecen-1-ol, 6,10-dimethyl-5,9-undecadien-2-one, and 2-pentanone) [60]. Wang et al. evaluate the idiopathic membranous nephropathy with 6 compounds found as significant [61]. Despite the small number, 23 chemical classes are included, where 3 are unique for this groups such as the dihydrofurans. One compound is found in all the nephrotic related studies (see Table S6): 4-heptanone.

### Other diseases

Applications with one or two studies are included in this group, such as children’s diseases, tuberculosis or coeliac disease. Cozzolino et al. used HS-SPME on urine samples to investigate children’s urine related to diseases [35, 62]. In one study, they aimed to find perturbations in the volatilome caused by overweight. The results showed 14 volatiles that distinguish between over-weight and normal children, from the more than 150 volatiles identified [62]. In a previous study, they compared the urinary profile of autistic children, finding that 3 volatiles under acidic conditions and 3 volatiles under alkaline conditions discriminated between groups [35]. Eshima et al. studied complex disorders, such as psychological disorders, due to the lack of quantitative diagnosis tools [63]. A multiple regression model led to 2 volatiles influenced by glucocorticoid signalling mechanism. SPME was used by Arasaradnam et al. to distinguish between coeliac patients and irritable bowel disease patients, 70 compounds were identified but with non-statistical significance [85]. Only one author used direct HS sampling, Banday et al. applied it to tuberculosis with 5 compounds found statistically significant [5]. Combination of the distinct studies increases the number of chemical classes found to 29 within this group. Where 2 chemical classes were not present in any other group,—cancer chemical exposure or nephrotic disease—(phenol ethers and pyrans). One compound is found in four of the five studies from other diseases (see Table S6): carvone.

## Discussion

In our report, we review the untargeted analysis of the urinary volatilome, in response to the demand for alternatives for more comprehensive and environmentally friendly compound extraction methods than the traditional ones. Among the different techniques available, solid phase microextraction (SPME) is simple, sensitive, robust, and an easily automated technique. The optimum SPME configuration depends on various factors, such as urine pre-analytical conditions, the fibre coating, time, and temperature of extraction. Data analysis also includes some crucial steps. For instance, normalization of urine, to which there is no clear consensus up to now, and compound identification, which depends on the available libraries of standard compounds and the information they provide (mass spectra alone or accompanied by RI). In summary, the HS–SPME–GC–MS technique to measure urine has been used in 26 studies with clinical applications finding/identifying/reporting utmost 227 volatiles. Other technologies like NTD-GC–MS showed similar results as SPME, reporting differences in the chemical classes found. Although the similarity in the technique, TD showed poor results, capturing a number of compounds distant of SPME or NTD results The use of hyphenated techniques, such as GCxGC-MS showed an increase in the urinary compounds detected with the identification of utmost 512 compounds [63].

Further developments are still needed in untargeted HS–SPME–GC–MS urinary analysis. Each SPME fibre covers only a narrow spectrum compared to the broad chemical spectrum of urine volatile compounds. In that sense, new tools like Arrow SPMEs, which have higher capacity and are more robust (are thicker and do not blend as easily as regular SPMEs), still have not been used in urinary samples. Another advantage of the Arrow SPME is that it also reduces the exposure time of the fibre [21, 86]. First studies with SPME Arrow-GC–MS in water samples have detected trace levels at the ng L−1 [87] concentrations. Concerning the newer fibres, newer developments have not yet been tested in urine samples like new coating materials which will be useful in all evaluated extraction techniques. As an example, we can mention new non-toxic coatings such as Carbon Nanotubes (CNTs), which have been applied to PAHs analysis from water samples, or the Sol–gel extraction phase, which has been proposed for polar compounds extractions in urine samples [86]. However, these techniques will be always dependent on the coatings affinity for the compounds of interest. Some authors recommend the use of more traditional extraction methods such as liquid–liquid extraction to broader the chemical classes obtained in a single analysis [88].

Resolving power between peaks depends on gas chromatography. Use of hyphenated techniques, like GCxGC-MS, have proven a remarkable increase in peak capacity (selectivity) and peak resolution. Coupling PAL-SPME Arrow extraction with GCxGC-MS promises an improvement on the number of compounds extracted and overall resolving power for urine samples.

Technology advances do not go hand in hand with sample processing advances. Compound identification is a bottleneck in metabolomics, the process is limited by the commercial availability of standard compounds. Confident identification of compounds should be done by standard comparison, however, not all compounds are commercially available or even their structure is not known properly. Some stablished methods, like specific gravity is not being used in urinary volatilomics, but are used in several longitudinal studies and by the World Anti-Doping Agency [79, 89, 90]. This method has the advantage of fewer cofounding effects and ease of measurement [91]. Although it is not an automatic method, it allows to correct sample concentration based on the hydration status of the individual. However, in urine volatilomics for biomedical applications, it is only used by one author which used it to select individuals between a specific range [60].

The major drawback when comparing results from different studies is the different nomenclature among the studies. The use of several libraries returns several options of name for the same chemical compound. To overcome this problem we used unique identifiers (IDs) like InChIKey instead the chemical name. The final biomedical untargeted urinary volatilome database (uBIOVOC DB) consists of 841 compounds all of them with a unique InChIKey and PubChem CID (Table S1). For re-usability of the UVDDB a part from chemical information (molecular formula and molecular weight), we provided several IDs. Up to 721 compounds have CAS number, but sixteen of them are used by more than one compounds. Databases IDs include KEGG (211 compounds), ChEBI (266 compounds), HMDB (387 compounds), LIPIDMAPS (152 compounds) and Drugbank ID (62 compounds). Moreover only 19 compounds had an entry in all the public databases retrieved, whereas 114 do not have any identifier associated.

Cancer is one of the pathologies most evaluated in the bibliography (*n* = 20), however the compound found more times was reported only 13 times (65% of the studies). Also, cancer is a very broad term for a wide range of tumours in different body localizations with different behaviours. Even though this heterogeneity, the enrichment analysis returned two significant pathways, both related to fatty acids metabolism: the fatty acids biosynthesis and the beta oxidation of very long chain fatty acids. None of the compounds found in at least half of the cancer studies analysed (2-pentanone, phenol, and dimethyl disulfide) was found relevant in the enrichment analysis. In the fatty acids metabolism are involved four volatiles, which are the lowest fatty acids: acetic acid (FA 2:0), hexanoic acid or caproic acid (FA 6:0), octanoic acid or caprylic acid (FA 8:0) and decanoic acid or capric acid (FA 10:0). Fatty acid metabolism supports tumorigenesis and disease progression through a range of processes including energy production (β-oxidation), membrane biosynthesis, energy storage and production, and generation of signalling intermediates [92]. Worth to mention the fact that most VOCs do not have HMDB or KEGG identifiers. From the 641 cancer-related compounds 422 do not have either of HMDB nor KEGG identifiers. Also, enrichment analysis is based upon a comparison to a set library. In our case, we used the small molecule pathway database (SMPDB) based on metabolites pathways. The improvement of SMPDB or even KEGG pathways databases with the incorporation of volatiles will have a very highly and positive impact in further studies.

For the exposure and nephrotic disease groups where all studies evaluated share one compound, hexanal, and 4-heptanone, respectively. The other diseases group also has a compound found in 80% of the articles, but it is a terpenoid related to food (carvone). The nephrotic disease group showed the lowest number of compounds (56 compounds), despite only half of the studies disclosed the complete list of compounds identified. In contrast, cancer group with the 80% of studies disclosing the complete list gathered 640 compounds, almost three times the number of compounds gathered in the other applications. Up to ten compounds are found in all the applications considered: 2-pentanone, 4-heptanone, hexanal, 2-heptanone, nonanal, 3-hexanone, benzaldehyde, Pyrroline, Dimethyl trisulfide, and Phenylmethylketone. However only three are found in more than 40% of all studies analysed: 2-pentanone, 4-heptanone, and hexanal. The 2-Pentanone has been associated to several diseases such as ulcerative colitis, non-alcoholic fatty liver disease, crohn's disease; and coeliac disease [75] [REF]. The 4-Heptanone has been also associated to several diseases such as kidney disease, perillyl alcohol administration for cancer treatment, and coeliac disease [75] [REF]. Hexanal is one of the most common aldehydes found in urine, as it is a major breakdown product of linoleic acid [75] [REF].

Among the high number of studies evaluating VOCs related to cancer there are initiatives of database creation for cancer [93, 94], however the websites are not maintained or the results are not specified by matrix. To overcome similar situations, we have included the biomedical untargeted urinary volatilome database as a supplementary material and is also available at Zenodo so that further reuse will be possible. Also, due the increase of studies evaluating urine and volatilome, the author’s intention is to update the biomedical untargeted urinary volatilome database every two years. The increase of evidence of urinary volatilome as a source of non-invasive and reliable testing, will promote its use in more biomedical applications. Therefore, this database will be of interest to a broad audience, ranging from basic researchers doing biomarkers discovery, to personalized medicine applications as it opens the floor for the development of predictive medicine devices, such as point-of-care or home testing devices [95].

## Conclusions

Our analysis compiled the largest database generated to date of urinary volatilomics data, with 841 compounds. Despite the high number of compounds reported, we have not restricted the inclusion of compounds by the level of identification or extraction technique. This is because a high level of confidence, comparison to reference standard or use of RI, is still limited in the bibliography. To overcome naming differences all compounds have been compared using a unique identificatory (the InChIKey in our case). However, the few compounds shared between studies show discrepancies in the results caused by different study designs or device coatings. The vast possibilities on the analysis technique contributes to the range of compounds obtained. In fact, less than 1% of all compounds is found in at least half of the studies evaluated, and no compound is reported in all the studies. Nonetheless, three compound are reported in all clinical groups: 2-pentanone, 4-heptanone and hexanal. Despite the different range of clinical applications, the comparison is usually done with healthy individuals (controls). The few compounds reported commonly reveals a need for standardization procedures, standardized analysis and reporting for the urinary volatilome. Nevertheless, the observed pattern of chemical classes found in the urinary volatilome will be helpful in deciding targeted compounds and methodology for further studies.

## Supplementary Information

**Additional file 1:** **Table S1.** The biomedical untargeted urinary volatilome database (uBIOVOC DB). **Table S2.** Summary of the sample collection conditions used by the 34 analysed studies. **Table S3.** Summary of the analytical conditions used by the 34 studies using them. **Table S4.** Summary of the GC-MS conditions used by the 34 analysed studies. **Table S5.** Summary of the applications analysed by the 34 analysed studies. **Table** **S6. **The urinary volatilome database by biomedical applications.

## Data Availability

The datasets generated during the current study are available in the Zenodo repository, link  10.5281/zenodo.6883341

## Abbreviations

HS: Headspace
TD: Thermal desorption
SPME: Solid-phase microextraction
NTD: Needle trap device
GC–MS: Gas chromatography – mass spectrometry
GCxGC-MS: Comprehensive gas chromatography – mass spectrometry
RI: Retention index
ID: Identified
IS: Internal standard
CAR: Carboxen
PDMS: Polydimethylsiloxane
DVB: Divinylbenzene
PA: Polyacrylate
CARX: Carbopack
LOWESS: Locally weighted scatterplot smoothing
PQN: Probabilistic quotient normalization
MSTUS: MS total useful signal
PG: Polyethylene glycol
BDPDP: 1,4-Bis(dimethylsiloxy)phenylene dimethyl polysiloxane
5DPDP: 5% Diphenyl—95% Dimethylpolysiloxane
6CPDP: 6% Cyanopropylphenyl – 94% Dimethylpolysiloxane
PP: Porus polymer
DMPS: Dimethylpolysiloxane
35PMP: (35%-Phenyl)-methylpolysiloxane
BC: Breast cancer
IDC: Beast invasive ductal carcinoma
CRC: Colorectal cancer
Pca: Prostate cancer
ccRCC: Clear cell renal cell carcinoma
RCC: Renal cell carcinoma
HNSCC: Head and Neck squamous cell carcinoma
HCN: Head and neck cancer
LC: Lung cancer
BdC: Bladder cancer
MsPGN: Mesangial proliferative glomerulonephritis
MCNS: Minimal change type nephrotic syndrome (MCNS)
iMN: Idiopathic membranous nephropathy
n.d.: Not disclosed
n.a.: Not applicable
CDK: Chronic kidney disease
ASDs: Autism spectrum disorders
OW/OB: Overweight/obesity
MS1: Metabolite standard level 1
RT: Retention time
HMDB: The human metabolome database
EI: Electron impact ionization
CNTs: Carbon nanotubes
PAHs: Polycyclic aromatic hydrocarbons
